# Severe Ethylene Glycol Toxicity: Multidisciplinary Management and Long-Term Renal Implications

**DOI:** 10.7759/cureus.76206

**Published:** 2024-12-22

**Authors:** Almothana Al-Kasabera, Zaid Alwarawrah, Love Kumar, Sarah Hatahet, Nabila Dawoud

**Affiliations:** 1 Internal Medicine, Griffin Hospital, Derby, USA

**Keywords:** acute kidney injury (aki), antifreeze ingestion, calcium oxalate crystals, chronic kidney disease (ckd), ethylene glycol poisoning, fomepizole, hemodialysis, metabolic acidosis, renal dysfunction

## Abstract

Ethylene glycol (C₂H₆O₂), a toxic alcohol commonly found in automotive antifreeze, de-icing solutions, and industrial coolants, can cause severe toxicity when ingested. Due to its sweet taste, it is often consumed accidentally or intentionally, leading to life-threatening consequences such as metabolic acidosis, acute kidney injury (AKI), and mortality. Prompt diagnosis and early treatment with antidotes such as fomepizole or ethanol, combined with hemodialysis, are essential in preventing severe outcomes. This report discusses the case of a 60-year-old male with a history of alcohol use disorder who presented with suspected ethylene glycol poisoning, confirmed by elevated serum levels (513 mg/dL). He received aggressive treatment including fomepizole and multiple hemodialysis sessions, which improved his acid-base status and renal function. Despite initial recovery, survivors of ethylene glycol poisoning remain at risk for developing chronic kidney disease (CKD). Prognostic factors such as severe metabolic acidosis, hyperkalemia, and neurological symptoms influence outcomes including developing CKD, with early intervention improving prognosis. This case emphasizes the importance of diagnosing ethylene glycol poisoning and initiating timely treatment to optimize outcomes and reduce long-term renal complications. Long-term follow-up is crucial for monitoring kidney function, as AKI survivors remain at higher risk for progressive renal decline.

## Introduction

Ethylene glycol (C_2_H_6_O_2_) is one of the toxic alcohols commonly found in products such as automotive antifreeze, de-icing solutions, and industrial coolants, as well as some cleaning agents and paints. It is a clear, colorless, sweet-tasting, and thick liquid at room temperature, though it is often dyed bright yellow-green when used in automotive antifreeze. Exposure to ethylene glycol can be highly dangerous if consumed in large quantities. The Environmental Protection Agency (EPA) recommends that children should not be exposed to more than 20 mg/L of ethylene glycol in drinking water for one day or 6 mg/L per day for 10 days. These limits highlight the potential health risks associated with ethylene glycol exposure, even at low concentrations, emphasizing the importance of strict regulation and monitoring to safeguard children's health. For adults, the EPA recommends a lifetime exposure of no more than 7 mg/L. The Scientific Committee on Food (SCF) set a tolerable daily intake (TDI) of 0.5 mg/day for ethylene glycol in food packaging materials. A toxic dose of ethylene glycol is considered to be more than 0.1 mL per kg of body weight. The minimum lethal dose for an adult is estimated to be 1 to 1.5 mL/kg (or around 100 mL). If not treated promptly, ethylene glycol exposure can lead to morbidity and mortality. Most exposures occur either through accidental ingestion - often because of its sweet taste - or intentional consumption, as seen in children, sometimes in suicide attempts, or as a substitute for alcohol. The toxic effects of ethylene glycol can vary in severity. Treatment generally involves supportive care and antidotes such as fomepizole or ethanol. These antidotes divert the enzymatic pathway to degrade ethanol rather than ethylene glycol, whose by-products are harmful to the body. In some cases, dialysis may also be needed to remove the toxin from the body.

Ethylene glycol is rapidly absorbed after ingestion and is initially eliminated at a rate proportional to its concentration, but when levels exceed 250 mg/dL, elimination becomes zero-order. If alcohol dehydrogenase is inhibited, elimination is significantly delayed.

Its metabolism begins in the stomach and mainly occurs in the liver, where it is first converted to glycolaldehyde by alcohol dehydrogenase and then to glycolic acid by aldehyde dehydrogenase. Glycolic acid leads to metabolic acidosis and is further converted to glyoxylic acid, which can form oxalic acid, a toxic compound that harms the kidneys. Thiamine, pyridoxine, and magnesium act as cofactors in the production of less harmful metabolites.

## Case presentation

A 60-year-old male with a history of alcohol use disorder, recurrent withdrawal seizures, ICU admissions, depression, anxiety, and gastroesophageal reflux disease presented unresponsive with labored breathing after being found in his truck, suspected to be intoxicated. He was hemodynamically stable in the field, initially confused and combative, but rapidly deteriorated in the ED despite multiple Narcan doses, requiring intubation for airway protection. On admission, he was afebrile, with a blood pressure of 150/108 and oxygen saturation of 98% on room air. Laboratory findings are demonstrated in Table 1.

**Table 1:** Labs on admission AST, aspartate transaminase; ALT, alanine transaminase

Test	Result	Reference range	Comments
ABG PH	7.05	7.35-7.45	Severe metabolic acidosis with delta gap of 0.34
ABG HCO_3_	<10 mmol/L	22-28 mmol/L	Significantly decreased
ABG PCO_2_	12.9 mmHg	35-45 mmHg	Decreased indicating respiratory compensation
ABG PO_2_	138.6 mmHg	80-100 mmHg	Elevated, likely due to hyperventilation
Serum osmolality	467 mOsm/kg	285-295 mOsm/kg	Elevated
Calculated osmolality	307 mOsm/kg	285-295 mOsm/kg	Within normal range
Osmolar gap	160	<10	Markedly elevated
Lactic acid	7.3 mmol/L	0.5-1.9 mmol/L	Elevated
Urinanalysis	3+ Calcium Oxalate Crystals	None	Diagnostic clue for ethylene glycol poisoning
Ketones	Negative	Negative	Normal
Sodium	149 mmol/L	136-145 mmol/L	Elevated
Magnesium	2.4 mg/dL	1.6-2.6 mg/dL	Within normal range
Phosphorus	5.0 mg/dL	2.4-5.19 mg/dL	Within normal range
Potassium	4.5 mmol/L	3.5-5.1 mmol/L	Within normal range
Chloride	120 mmol/L	98-107 mmol/L	Elevated
Glucose	103 mg/dL	74-106 mg/dL	Within normal range
Creatinine	0.82 mg/dL	0.73-1.118 mg/dL	Within normal range
Blood urea nitrogen	9.8 mg/dL	9-23 mg/dL	Within normal range
AST	41 U/L	0.0-33.9 U/L	Elevated
ALT	20 U/L	10-49 U/L	Within normal range
Alkaline phosphatase	67 U/L	46-110 U/L	Within normal range
Albumin	4.6 g/dL	3.2-4.8 g/dL	Within normal range
White blood cell count	8.4/µL	4.8-10.8/µL	Within normal range
Hemoglobin	13.8 g/dL	14-18 g/dL (male)	Slightly below normal range
Mean corpuscular volume	103 fL	80-94 fL	Elevated indicating macrocytic anemia
Platelets	174/µL	140-440/µL	Within normal range

Imaging, including CT of the head, neck, abdomen, and pelvis, was notable for fatty liver infiltration and a partially imaged lingular infiltrate; chest X-ray was unremarkable. Ethylene glycol toxicity was suspected, prompting testing for ethylene glycol and methanol, empiric fomepizole administration, and consultation with nephrology and poison control. The patient remained critically ill in the ICU on mechanical ventilation with worsening acidemia (pH 7.05 at admission, then 6.9 after 3 hours, and 6.7 after 4 more hours), requiring a bicarbonate drip, IV fluids, and hemodynamic monitoring; the patient had not received dialysis yet at that point because ethylene glycol result did not come back. Ethylene glycol came back at 513 mg/dL, and methanol was negative. After ethylene glycol result came back, the plan included 9 hours of dialysis with fomepizole redosing every 4 hours after the patient received the loading dose empirically, with rechecking ethylene glycol levels every 6-12 hours and interim monitoring for kinetics. It was suspected that he would require further dialysis from the standpoint of acute kidney injury (AKI). Aggressive management was initiated to address severe metabolic derangements, suspected sepsis or aspiration pneumonia treated empirically with antibiotics pending sputum and blood culture results, and complications such as AKI, hyperkalemia, hypertension, and hyperglycemia. The pH improved to 7.47 after the first session of dialysis. The patient continued to have dialysis without fluid removal and fomepizole until his ethylene glycol levels were below 20 mg/dL. His ethylene glycol level was 15 mg/dL on day 2 and undetected on day 3. However, his creatinine gradually increased to 8.37 on day 8, and his urine output decreased gradually to 155 mL/24 hours on day 7. His blood pressure became high and hard to control. Accordingly, the patient continued to have more dialysis sessions, this time with around 3-4 liters of fluid removed on day 6 and day 8. The patient was extubated on day 8 and admitted that he drank antifreeze because he was depressed and could not quit drinking alcohol. His urine output started picking up by day 10; therefore, another dialysis was performed on day 10 without fluid removal. The patient's urine output and kidney function continued to improve. He did not need further dialysis, and the dialysis catheter was removed on day 15. Of note, the patient's urine output was more than 900 mL, and creatinine was 1.63 on the day of discharge (day 21).

## Discussion

Ethylene glycol poisoning is a severe medical emergency that demands prompt diagnosis and treatment to mitigate the risk of devastating outcomes such as metabolic acidosis, AKI, and mortality. Common early symptoms include nausea, vomiting, headache, and confusion. We cannot identify the situation just by the symptoms initially as they are non-specific, and we would need either history or additional labs to have an initial suspicion.

The clinical progression of ethylene glycol toxicity is typically categorized into three distinct phases: the initial phase of central nervous system (CNS) depression, followed by cardiopulmonary complications associated with profound metabolic acidosis including irregular heart rhythms, hypotension, and respiratory failure [1]. The toxic metabolites of ethylene glycol, particularly glycolic acid, contribute to severe metabolic acidosis, which can destabilize both cardiac and respiratory functions, and, finally, renal impairment caused by oxalic acid deposition and crystal formation in the renal tubules. Acute tubular necrosis is the type of renal tubule injury that occurs in cases of ethylene glycol poisoning. This injury is primarily due to the deposition of calcium oxalate crystals within the tubular epithelial cells, leading to widespread necrosis of the tubular epithelium, particularly in the proximal tubules [2-4]. In addition to acute tubular necrosis, ethylene glycol poisoning can cause direct cellular toxicity due to metabolites such as glycolaldehyde and glyoxylate, which lead to ATP depletion and membrane phospholipid degradation in renal tubular cells. Furthermore, the deposition of calcium oxalate crystals within the renal tubules can cause mitochondrial dysfunction and increased reactive oxygen species, contributing to cellular injury and necrosis [2,3,5]. Early recognition and intervention are critical to improving prognosis and reducing long-term complications [6,7].

Prognostic factors significantly influence the clinical outcomes of ethylene glycol poisoning. Hylander and Kjellstrand identified severe metabolic acidosis, hyperkalemia, and neurological symptoms such as seizures and coma upon presentation as markers of poor prognosis. Their study demonstrated that patients exhibiting these features have an elevated risk of mortality. Additionally, the detection of calcium oxalate crystals in the urine serves as an important diagnostic clue and a marker of the severity of poisoning [8]. These findings underscore the need for rapid assessment and aggressive treatment to reverse the toxic effects and prevent further deterioration.

The treatment of ethylene glycol poisoning involves administering antidotes such as fomepizole or ethanol, which act by inhibiting alcohol dehydrogenase, the enzyme responsible for metabolizing ethylene glycol into toxic metabolites. Fomepizole is generally preferred due to its superior safety profile and effectiveness, especially when administered early in the poisoning course. For patients with significant complications, such as severe acidosis, renal failure, or markedly elevated ethylene glycol levels, hemodialysis is a critical intervention. Hemodialysis effectively removes ethylene glycol and its toxic metabolites while correcting metabolic derangements, making it an essential component of the treatment protocol in severe cases [6,8,9].

Prompt and accurate diagnosis is crucial for effective management. While serum ethylene glycol levels provide definitive confirmation, they are often unavailable in acute settings. As a result, clinicians rely on surrogate markers such as a widened osmolar gap, anion gap metabolic acidosis, and elevated lactate with a “lactate gap” to guide diagnosis. The presence of these findings should prompt the immediate initiation of antidotal therapy and consideration of hemodialysis [1,7]. Early recognition and timely intervention remain the key to reducing morbidity and mortality in cases of ethylene glycol poisoning.

The prognosis for renal recovery in ethylene glycol poisoning is generally favorable if treatment is initiated early. A systematic review and meta-analysis reported that kidney recovery occurred in 64.7-96.3% of patients with ethylene glycol poisoning at hospital discharge, with only 2-3.7% requiring ongoing dialysis [10]. These findings highlight the importance of early diagnosis and intervention in improving renal outcomes. Case reports have documented complete recovery of kidney function even in severe cases of ethylene glycol poisoning. For instance, a 36-year-old man with a history of severe ethylene glycol poisoning and an extremely high ethylene glycol concentration survived without persistent kidney failure following sustained hemodialysis and ethanol infusion [11]. Similarly, a 90-year-old woman recovered completely from profound metabolic acidosis despite a delay in diagnosis and treatment [12].

Additionally, studies on AKI survivors, such as the one by Sawhney et al., indicate that patients who have experienced AKI are at a higher risk for subsequent renal decline compared to those without AKI [13]. This is relevant because ethylene glycol poisoning often results in AKI, and the long-term risk of kidney function deterioration persists.

For our case, the patient's kidney function was improving but did not fully recover by the time of discharge. At that time, it could not be determined if the patient would develop a component of chronic kidney disease, as further and longer follow-up is needed. Even after initial recovery, patients with a history of ethylene glycol poisoning are at an increased risk for long-term kidney complications.

Furthermore, Horne et al. demonstrated that kidney disease progression is significantly increased in patients with a history of AKI, with 30% of the exposed group showing progression at five years compared to 7% of the non-exposed group [14]. This underscores the long-term vulnerability of kidneys post-AKI, including those affected by ethylene glycol poisoning.

## Conclusions

The prognosis of ethylene glycol poisoning depends on the severity of metabolic acidosis and the timeliness of treatment. Early administration of fomepizole and hemodialysis are critical interventions that can significantly improve outcomes. Clinicians should be vigilant in recognizing the clinical and laboratory signs of ethylene glycol poisoning to initiate prompt and appropriate treatment. Patients with a history of ethylene glycol poisoning are at a higher risk for rapid worsening of kidney function over time, even after initial recovery, compared to individuals without such a history. This necessitates careful long-term monitoring, management, and addressing other risk factors such as hypertension and diabetes to mitigate further renal decline. Additionally, the recovery of kidney function in ethylene glycol poisoning is highly dependent on the prompt administration of fomepizole and hemodialysis. Early intervention can significantly improve renal outcomes and reduce the need for long-term dialysis. Clinicians should remain vigilant in recognizing the clues to the diagnosis of ethylene glycol poisoning and initiating appropriate treatment without delay to optimize patient outcomes.
